# How to diagnose GERC more effectively: reflections on post-reflux swallow-induced peristaltic wave index and mean nocturnal baseline impedance

**DOI:** 10.1186/s12890-024-03080-z

**Published:** 2024-06-05

**Authors:** Bingxian Sha, Wanzhen Li, Haodong Bai, Tongyangzi Zhang, Shengyuan Wang, Wenbo Shi, Siwan Wen, Li Yu, Xianghuai Xu

**Affiliations:** Department of Pulmonary and Critical Care Medicine, Tongji Hospital, School of Medicine, Tongji University, Shanghai, China

**Keywords:** Chronic cough, Gastroesophageal reflux-related chronic cough, Post-reflux swallow-induced peristaltic wave index, Mean nocturnal baseline impedance

## Abstract

**Introduction:**

Post-reflux swallow-induced peristaltic wave index **(**PSPWI) and mean nocturnal baseline impedance (MNBI) are novel parameters reflect esophageal clearance capacity and mucosal integrity. They hold potential in aiding the recognition of gastroesophageal reflux-induced chronic cough (GERC). Our study aims to investigate their diagnostic value in GERC.

**Methods:**

This study included patients suspected GERC. General information and relevant laboratory examinations were collected, and final diagnosis were determined following guidelines for chronic cough. The parameters of multichannel intraluminal impedance-pH monitoring (MII-pH) in patients were analyzed and compared to explore their diagnostic value in GERC.

**Results:**

A total of 186 patients were enrolled in this study. The diagnostic value of PSPWI for GERC was significant, with the area under the working curve (AUC) of 0.757 and a cutoff value of 39.4%, which was not statistically different from that of acid exposure time (AET) (*p* > 0.05). The combined diagnostic value of AET > 4.4% and PSPWI < 39.4% was superior to using AET > 4.4% alone (*p* < 0.05). Additionally, MNBI and distal MNBI also contributed to the diagnosis of GERC, with AUC values of 0.639 and 0.624, respectively. AET > 4.4% or PSPWI < 39.4% is associated with a 44% reduction in missed diagnoses of non-acid GERC compared to AET > 6.0% or symptom association probability (SAP) ≥ 95%, and may be more favorable for identifying GERC.

**Conclusion:**

The diagnostic value of PSPWI for GERC is comparable to that of AET. Combining PSPWI < 39.4% or AET > 4.4% can improve the diagnostic efficiency by reducing the risk of missed diagnoses in cases where non-acid reflux is predominant. Distal MNBI and MNBI can serve as secondary reference indices in the diagnosis of GERC.

## Introduction

Cough is the most common complaint among patients seeking care at primary healthcare facilities and respiratory specialty clinics. Chronic cough is typically defined as a cough lasting more than 8 weeks [1–3]. Gastroesophageal reflux-related cough (GERC) is one of the common causes of chronic cough [4–6], accounting for 10-40% of the etiology of chronic cough, with an increasing trend over the years. However, the relationship between chronic cough and reflux is complex, and the diagnosis of GERC poses significant challenges. Currently, multichannel intraluminal impedance-pH monitoring (MII-pH) is an important adjunctive test for the diagnosis of GERC as stated in relevant clinical guidelines [4, 7]. It is frequently utilized to establish the temporal association between reflux and cough, while also evaluating the severity of reflux in subjects. MII-pH demonstrates good diagnostic performance in the diagnosis of GERC. During this assessment process, parameters such as acid exposure time (AET), symptom association probability (SAP) and symptom index (SI) play a crucial role [5, 8]. AET is a reliable parameter for identifying acid reflux. According to the global consensus on gastroesophageal reflux disease, AET > 6% is considered abnormal, but AET values between 4% and 6% require further analysis with other parameters [8]. At the same time, AET cannot effectively identify non-acid reflux-related gastroesophageal reflux cough, which limits its clinical application [9]. SI and SAP indicate the temporal relationship between cough and reflux, independent of the acidity of the reflux material. However, due to their subjective nature and high compliance requirements [10], patients often find it difficult to accurately record the actual occurrence of cough events, leading to an overestimation of their clinical significance [11]. Nowadays, there is a need for more objective and accurate diagnostic parameters to overcome these limitations.

Post-reflux swallow-induced peristaltic wave index (PSPWI) and mean nocturnal baseline impedance (MNBI) are parameters derived from monitoring techniques based on MII-pH in recent years. PSPWI reflects the esophageal clearance ability, while MNBI reflects the electrical conductivity and mucosal integrity of the esophagus and is correlated with esophageal tissue pathological changes [12]. Current research indicates that impaired esophageal clearance function can cause incomplete reflux clearance, and the erosion of reflux on the esophageal mucosa can cause a decrease in esophageal baseline impedance. MNBI is significantly lower in patients with gastroesophageal reflux disease (GERD) compared to healthy subjects and those with functional heartburn (FH) [13]. PSPWI is used to evaluate the degree of impaired esophageal clearance function in GERD patients, and it is significantly lower in patients with non-erosive reflux disease (NERD) than in those with FH and normal subjects [14]. In recent years, studies have shown that the application of PSPWI and MNBI can improve the diagnostic efficacy of MII-pH for GERD [12, 15]. We speculate that they may also have predictive value for GERC in the chronic cough population. Therefore, this study retrospectively analyzed the clinical and laboratory data of suspected GERC patients who visited our department, aiming to explore the diagnostic value of PSPWI and MNBI for GERC.

## Methods

### Patients

This is a retrospective observational study. The data was obtained from the Chronic Cough Database of the Department of Respiratory and Critical Care Medicine, Tongji Hospital, Tongji University, between March 2018 and January 2023. After a detailed analysis of the medical history, comprehensive physical examination, and capsaicin cough sensitivity testing, the etiology of chronic cough was determined based on laboratory examinations including chest X-ray or chest CT, pulmonary function tests, bronchial provocation tests, induced sputum cytology, MII-pH monitoring, etc. The diagnosis of GERC was based on the international consensus proposed by the American College of Chest Physicians and the Chinese Medical Association in conjunction with the international consensus on GERD [4, 8, 16]. When the following criteria were met, GERC was considered: [1] chronic cough, with daytime cough being common; [2] AET > 6.0%, SI ≥ 50% and/or SAP ≥ 95% on MII-pH monitoring [3], cough improved significantly or disappeared after step-by-step anti-reflux treatment (cough symptom score decreased by > 50%). If AET > 6.0% and acid SI ≥ 50% and/or acid SAP ≥ 95%, the patient was diagnosed with acid GERC. If the gastric acid reflux was negative, AET ≤ 6.0%, but non-acid SI ≥ 50% and/or non-acid SAP ≥ 95%, and the patient responded to step-by-step anti-reflux treatment [17], then the diagnosis was non-acid GERC. In some patients, their cough was partially improved after receiving treatment for chronic cough caused by factors other than GERC. However, their cough completely disappeared after receiving combined anti-reflux treatment. As a result, the final diagnosis was determined as GERC combined with other etiologies.

### MII-pH monitoring

All patients who underwent MII-pH monitoring had abstained from proton pump inhibitors (PPI) for at least 2 weeks. The position of the lower esophageal sphincter was determined using esophageal manometry (Solar GI, Medical Measurement System B.V., Netherlands). A combined MII-pH catheter with a diameter of 2.1 mm was inserted through the nasal passage into the patient’s esophagus, including six impedance channel sensors (K6011-E10632, Unisensor, Attikon, Switzerland) and an antimony pH electrode (819,100, Medical Measurement System BV, Enschede, Netherlands). The impedance channel sensors were positioned at 3, 5, 7, 9, 15, and 17 cm above the lower esophageal sphincter, while the pH electrode was placed approximately 5 cm proximal to the lower esophageal sphincter. The catheter was connected to a portable data logger (Ohmega, Medical Measurement System BV) to collect data from all seven channels. Patients were instructed to maintain their normal lifestyle and habits during the monitoring period and to record cough episodes, meal times, and changes in body position on a diary card and pressing an event marker button in the portable data logger during monitoring.

### MII-pH monitoring data analysis

The data was automatically analyzed using specific software (MMS database, v8.7). Reflux events were manually reviewed and classified based on impedance values into gas, liquid, and mixed reflux events. They were further categorized as acid reflux events (pH < 4.0), weak acid reflux events (pH 4.0–7.0), and weak alkaline reflux events (pH > 7.0). When cough events recorded in the diary card appeared within a 2-minute window of reflux events, they were considered reflux-related and expressed as Symptom Association Probability (SAP) [18]. The proportion of reflux-related cough events to the total number of cough events was represented as Symptom Index (SI) [19]. AET represents the percentage of time with esophageal pH < 4.0 during the entire monitoring period [8]. DeMeester score [20] is a weighted score consisting of six parameters: total reflux time, upright reflux time, supine reflux time, total number of reflux episodes, number of reflux episodes lasting longer than 5 min, and the longest duration of reflux episode. A post-reflux swallow-induced peristaltic wave (PSPW) has been defined as an antegrade 50% drop in impedance originating in the proximal esophagus within 30s after the end of a reflux episode and reaching the distal esophageal lumen [21]. The PSPWI is calculated by dividing the number of PSPW events by the total number of reflux events [22]. The MNBI is obtained by calculating the average impedance values of six channels during three 10-minute periods at night (approximately at 1:00 am, 2:00 am, and 3:00 am). The proximal MNBI represents the average impedance values of two channels located 15 cm and 17 cm above the lower esophageal sphincter (LES), while the distal MNBI represents the average impedance values of four channels located 3 cm, 5 cm, 7 cm, and 9 cm above the LES [23].

### Other laboratory investigations

According to the modified capsaicin cough challenge test proposed by Fujimura et al. [24], cough sensitivity was measured by determining the inhalation of the lowest concentrations of capsaicin solution that induced 2 and 5 coughs, referred to as the cough thresholds C2 and C5, respectively, which represent the subjects’ cough sensitivity. Induced sputum cytology examination [25] was performed, and pulmonary function tests and bronchial provocation tests were conducted following the guidelines established by the American Thoracic Society [26] and the Chinese Medical Association [27].

### Statistical analysis

Normally distributed data are presented as mean ± standard deviation (χ2 ± SD), while skewed data are presented as median (interquartile range). The diagnostic value of various impedance parameters for GERC was assessed by plotting receiver operating characteristic (ROC) curves, and statistical differences between ROC curves were compared using the Delong test. SPSS 27.0 and Medcalc 20.0 were used for statistical analysis. *P* < 0.05 was considered statistically significant.

## Results

### Basic information

Between March 2018 and January 2023, a total of 683 patients with chronic cough were seen at the Department of Respiratory and Critical Care Medicine, Tongji Hospital, Tongji University. Among them, 204 patients underwent MII-pH monitoring for suspected GERC. Eighteen patients were excluded due to incomplete follow-up data, leaving 186 patients with chronic cough included in this study. According to the aforementioned diagnostic and treatment process for chronic cough, 132 patients were diagnosed with GERC, including 82 patients with acid GERC and 50 patients with non-acid GERC. Among them, 115 patients had GERC as the sole cause of their chronic cough, while 17 patients had GERC combined with other common causes of chronic cough, including 7 cases with chronic viral asthma (CVA), 5 cases with allergic cough (AC), 2 cases with eosinophilic bronchitis (EB), and 3 cases with upper airway cough syndrome (UACS). Fifty-four patients were excluded from GERC and considered to have chronic cough caused by other reasons, including 8 cases of UACS, 8 cases of AC, 15 cases of CVA, 10 cases of EB, 4 cases of acute cough and ACE inhibitor-related cough, 4 cases of CVA combined with EB, 3 cases of CVA combined with UACS, and 2 cases of unknown causes of chronic cough. General information of all patients included in the study is shown in Table 1. The baseline differences among the groups were not statistically significant.

**Table 1:** General clinical characteristics of patients

Variables	GERC (*n* = 132)	Non-GERC (*n* = 54)
**Age(years)**	51.00(26.75)	47.00(25.25)
**Gender (F/M)**	71/61	31/23
**Course of cough (m)**	16.00 (18.00)	20.00 (17.00)
**Cough symptom score**		
**Daytime**	3.00 (1.00)	3.00 (1.00)
**Nighttime**	1.00 (1.00)	2.00 (1.00)
**Cough sensitivity**		
**C2(µmol/L)**	0.49 (1.46)	0.49 (0.49)
**C5(µmol/L)**	1.95 (7.31)	0.49 (0.49)
**Lung function (%, x ± s)**		
**FEV1 predicted (%)**	100.30 ± 12.78	102.16 ± 12.03
**FVC predicted (%)**	98.11 ± 19.87	96.59 ± 10.23
**FEV1/FVC%**	86.22 ± 14.22	90.20 ± 9.78

C2, capsaicin solution concentration with ≥ 2 coughs; C5, capsaicin solution concentration for ≥ 5 coughs; FEV1, forced expiratory volume in 1s; FVC, forced vital capacity

### Comparison of MII-pH parameters between GERC and Non-GERC groups

We included a total of 186 patients in our study, divided into the GERC group and the non-GERC group. We collected and summarized the MII-pH parameters for both groups in Table 2. Compared to the non-GERC group, the GERC group had higher AET and DeMeester scores (Z = -7.220, *p* < 0.001; Z = -7.225, *p* < 0.001), as well as higher SAP, SI, and reflux episodes (Z = -3.536, *p* < 0.001; Z = -3.477, *p* < 0.001; Z = -2.027, *p* = 0.043). There were differences between the two groups in terms of MNBI and distal MNBI, with GERC patients showing lower values compared to non-GERC patients (Z = -2.658, *p* = 0.008; Z = -2.969, *p* = 0.003). Additionally, there was a significant difference in PSPWI between the two groups, with GERC group exhibiting lower values than the non-GERC group (Z = -5.493, *p* < 0.001).

**Table 2:** Comparison of different variables of MII-pH between three groups

Variables	GERC (*n* = 132)	Non-GERC (*n* = 54)
**AET (%)**	5.85(8.80) *	0.95(2.12)
**DeMeester score**	20.22(27.74) *	3.43 (6.42)
**SAP (%)**	70.80(92.05) *	0.00 (62.95)
**Acid SAP (%)**	0.00(77.88)	0.00 (0.00)
**Non-acid SAP (%)**	0.00(91.28) *	0.00 (0.00)
**SI (%)**	12.50(33.30) *	0.00 (14.30)
**Acid SI (%)**	0.00(9.78) *	0.00 (0.00)
**Non-acid SI (%)**	0.00(16.27) *	0.00 (0.55)
**Reflux episodes(n)**	65.50(67.25) *	57.00 (40.00)
**Acidic reflux (n)**	26.00(28.00) *	14.50 (24.00)
**Non-acidic reflux (n)**	36.50(52.25)	40.50 (34.25)
**Weakly acidic reflux (n)**	28.50(41.75)	23.00 (26.75)
**Weakly alkaline reflux (n)**	5.00(15.75) *	10.00 (14.50)
**Gas reflux (n)**	13.95(18.58)	14.50 (21.23)
**Liquid reflux (n)**	14.75(18.88) *	12.50 (14.60)
**Mixed reflux (n)**	40.00(45.75) *	25.00 (28.25)
**MNBI(Ω)**	2277.64(862.58) *	2446.59 (655.82)
**Proximal MNBI(Ω)**	2400.67(806.92)	2386.00(711.96)
**Distal MNBI(Ω)**	2233.96(1011.72) *	2504.04(685.67)
**PSPWI**	36.44(7.03) *	43.83 (11.80)

^*^: *p* < 0.05

### Exploring the Diagnostic Value of MNBI and PSPWI for GERC

#### Comparison of diagnostic value of MII-pH parameters for GERC

**Fig. 1:**
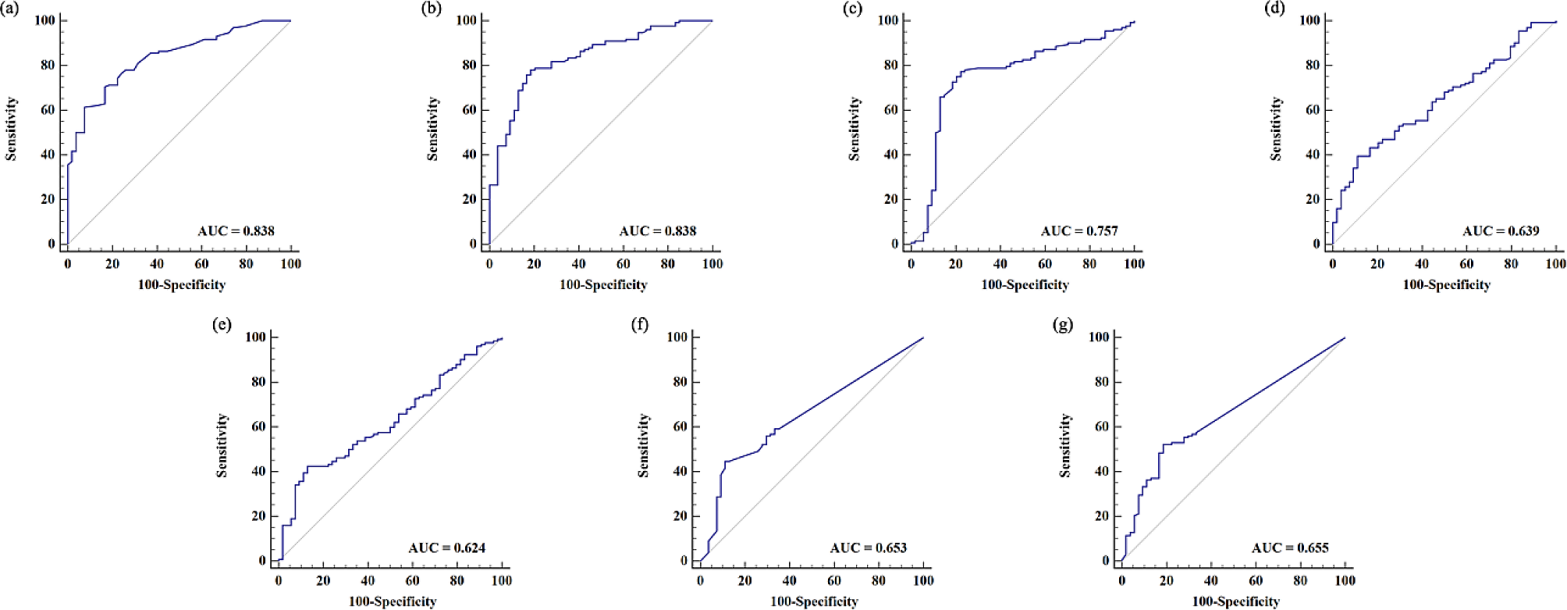
The diagnostic value of AET, DeMeester score, PSPWI, Distal MNBI, MNBI, SI and SAP for GERC. **(a)** AET; **(b)** DeMeester score; **(c)** PSPWI; **(d)** Distal MNBI; **(e)** MNBI; **(f)** SI; **(g)** SAP;

We examined the diagnostic value of classic MII-pH monitoring parameters, including AET, DeMeester score, SI, and SAP for GERC. We compared them with PSPWI, distal MNBI, and MNBI, and summarized the ROC curves in Fig. 1. AET and DeMeester score showed high diagnostic value for GERC, with AUCs of 0.838 for both parameters. The optimal cutoff value for AET was found to be 4.4%, yielding the highest Youden’s index. SI and SAP showed some diagnostic value for GERC as well, with AUCs of 0.655 and 0.653, respectively. Distal MNBI and MNBI had moderate diagnostic value for GERC, with AUCs of 0.639 and 0.624, respectively. However, proximal MNBI had no diagnostic value for GERC, with an AUC of 0.500.

The PSPWI showed a smaller AUC compared to AET and DeMeester score for GERC diagnosis. However, the differences were not statistically significant (AUC_ROC_ = 0.838 vs. AUC_ROC_ = 0.757, *p* = 0.102; AUC_ROC_ = 0.838 vs. AUC_ROC_ = 0.757, *p* = 0.089). Nevertheless, it was significantly superior to SI and SAP (AUC_ROC_ = 0.757 vs. AUC_ROC_ = 0.653, *p* = 0.014; AUC_ROC_ = 0.757 vs. AUC_ROC_ = 0.655, *p* = 0.027). When the cutoff value was set to 39.4%, the maximal Youden index was 0.543, with a sensitivity of 76.52% and specificity of 77.78%. Distal MNBI and MNBI had some diagnostic value for GERC, with cutoff values of 2004.75Ω and 2102.50Ω, respectively. However, they were weaker predictors compared to AET and DeMeester score, and showed no statistically significant difference compared to SI and SAP (DeLong’s test, all ps > 0.05).

#### Investigation of the Diagnostic Value of MNBI, Distal MNBI combined with PSPWI and AET for GERC

The above results indicate that PSPWI, MNBI, and distal MNBI have diagnostic value for GERC. We defined AET > 4.4% as the diagnostic criterion (I) for GERC, PSPWI < 39.4% as the diagnostic criterion (II), Distal MNBI < 2004.75Ω as the diagnostic criterion (III), and MNBI < 2102.5Ω as the diagnostic criterion (IV). Table 3 shows the diagnostic value of GERC when combining and comparing these parameters.

Both (I) or (II), (I) or (III), (I) or (IV), (II) or (III), and (II) or (IV) demonstrate good diagnostic value for GERC, and exhibit significant advantages over (V) SAP ≥ 95% and (VI) SI ≥ 50% (DeLong test: all ps < 0.05). Compared to (I) with AET > 4.4%, there was no statistically significant difference in the diagnostic value of GERC prediction among (I) or (III), (I) or (IV), (II) or (III), and (II) or (IV) (DeLong test: *p* = 0.728; *p* = 0.914; *p* = 0.692; *p* = 0.632). However, (I)AET > 4.4% or (II)PSPWI < 39.4% demonstrated a better diagnostic value for GERC prediction, with an AUC of 0.819 (DeLong test: *p* = 0.037).

**Table 3:** Comparison of diagnostic value of several items for GERC

	AUC	Sensitivity (%)	Specificity (%)	Positive predictive value	Negative predictive value	Positive likelihood ratio	Negative likelihood ratio	Kappa
**(I) AET > 4.4**%	0.770	61.36	92.59	95.29	49.50	8.28	0.42	0.429
**(II) PSPWI < 39.4%**	0.771	76.52	77.78	89.38	57.53	3.44	0.30	0.492
**(III) MNBI < 2102.50Ω**	0.630	40.91	85.19	87.10	37.10	2.76	0.69	0.189
**(IV) Distal MNBI** **< 2004.75Ω**	0.634	37.88	88.89	89.29	36.92	3.41	0.70	0.189
**(I) and (II)**	0.699	41.67	98.15	98.21	40.77	22.52	0.59	0.281
**(I) or (II)**	0.819	91.67	72.22	88.97	78.00	3.30	0.12	0.653
**(I) and (III)**	0.618	27.27	96.30	94.74	35.14	7.37	0.76	0.156
**(I) or (III)**	0.734	68.94	77.78	88.35	50.60	3.10	0.40	0.403
**(I) and (IV)**	0.631	28.03	98.15	97.37	35.81	15.15	0.73	0.173
**(I) or (IV)**	0.750	66.67	83.33	90.72	50.56	4.00	0.40	0.420
**(II) and (III)**	0.620	29.55	94.44	92.86	35.42	5.31	0.75	0.161
**(II) or (III)**	0.769	87.12	66.67	86.47	67.92	2.61	0.19	0.541
**(II) and (IV)**	0.614	26.52	96.30	94.59	34.90	7.17	0.76	0.150
**(II) or (IV)**	0.765	86.36	66.67	86.36	66.67	2.59	0.20	0.530
**(V) SAP ≥ 95%**	0.571	19.70	94.44	89.66	32.48	3.54	0.85	0.106
**(VI) SI ≥ 50%**	0.531	13.64	92.59	81.82	30.49	1.84	0.53	0.039
**X** ^**2**^		541.386	83.507	17.934	95.252			
**P value**		0.001	0.001	0.266	0.001			

#### Investigating the advantages of employing AET > 4.4% or PSPWI < 39.4% for diagnosing GERC

**Fig. 2:**
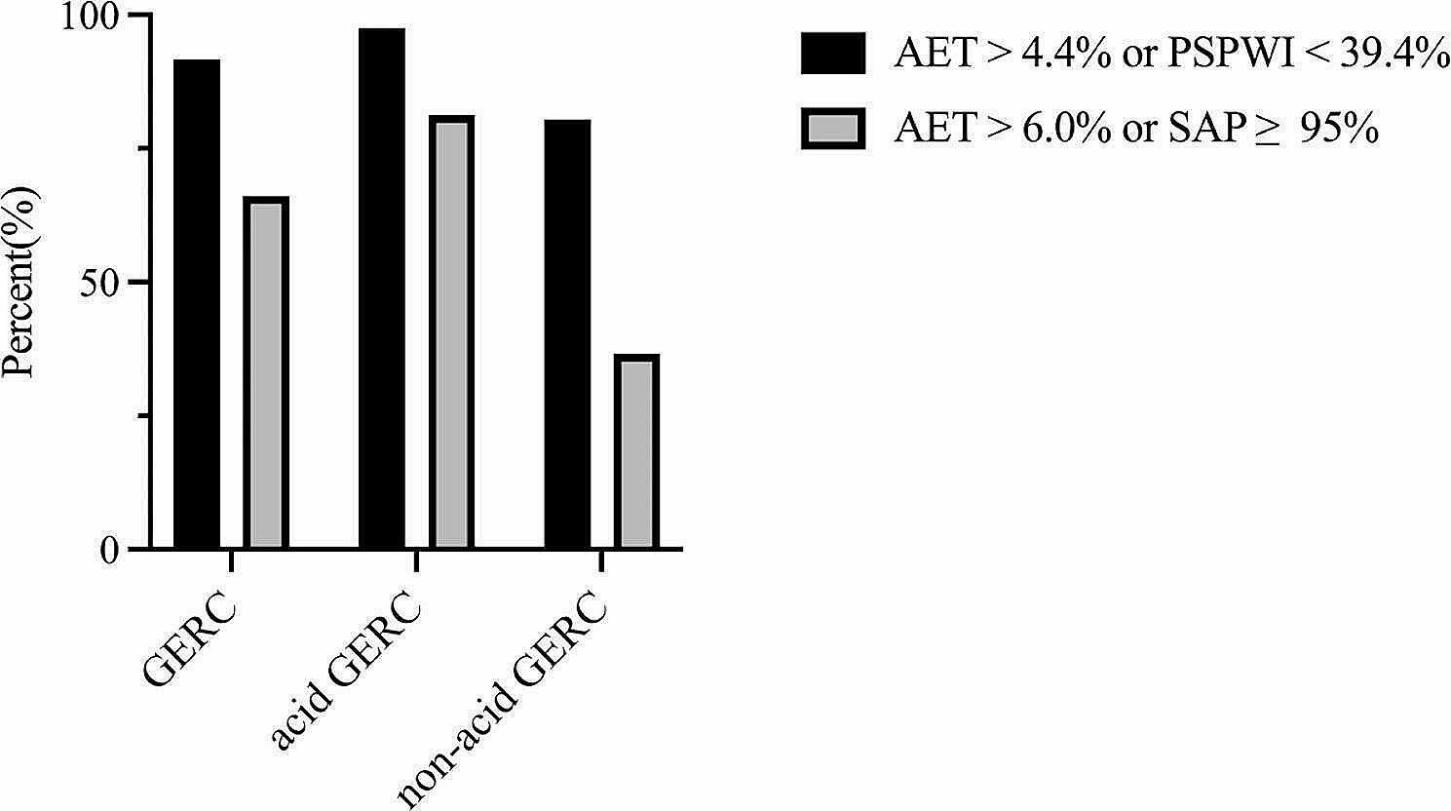
The diagnostic rates of the two diagnostic parameters in different types of GERC

According to the current international guidelines and expert consensus [4, 8, 16], the parameter for the initial diagnosis of GERC through laboratory testing presently employed by clinicians is a combination of AET > 6.0% or SAP ≥ 95%. We separately analyzed the composition of different types of GERC patients diagnosed using (I)AET > 4.4% or (II)PSPWI < 39.4% and AET > 6.0% or SAP ≥ 95%, and plotted the results in Fig. 2. Among the 186 patients suspected GERC included in the study, 132 were definitively diagnosed with GERC. Among these, 91.74% of patients could be diagnosed using AET > 4.4% or PSPWI < 39.4%, while AET > 6.0% or SAP ≥ 95% could only identify 66.12% of GERC patients. Both parameters resulted in a diagnosis rate exceeding 80% for the 82 acid GERC patients. However, for the remaining 52 non-acid GERC patients, AET > 4.4% or PSPWI < 39.4% maintained a diagnosis rate of 80.49%, which was an increase of approximately 44% compared to the 36.59% diagnosis rate achieved by AET > 6.0% or SAP ≥ 95%. Therefore, we recommend using AET > 4.4% or PSPWI < 39.4% as the diagnostic criterion for identifying GERC patients to effectively avoid missed diagnoses.

## Discussion

This study reveals that patients in the GERC group exhibit significantly lower levels of PSPWI, MNBI, and distal MNBI compared to non-GERC patients. AET demonstrated a higher diagnostic value for GERC, and PSPWI also had a certain diagnostic value similar to AET, notably superior to SI, SAP, MNBI, and distal MNBI. MNBI and distal MNBI had limited diagnostic efficacy for GERC and showed no statistically significant difference compared to SI and SAP. The combination of PSPWI and AET provided a higher diagnostic value for GERC, surpassing the diagnostic performance of AET alone, effectively reducing misdiagnosis. This may be potentially associated with improving recognition of the non-acid GERC.

GERD encompasses symptoms both within and outside the esophagus, while GERC represents the specific extraintestinal manifestations of GERD [28, 29]. Two different mechanisms may underlie the relationship between reflux and cough: the reflux pathway leading to micro-aspiration and the reflex pathway triggering vagally mediated airway reactions [30]. When GERD induces cough, approximately 75% of patients may lack obvious gastrointestinal symptoms [31], making it challenging to preliminarily distinguish GERC based on clinical manifestations alone. MII-pH monitoring is the most sensitive and specific auxiliary examination for diagnosing GERC. By monitoring parameters such as AET, SI, and SAP, the severity of reflux is evaluated alongside its correlation with coughing [32]. AET is a parameter associated with the severity of acid exposure in subjects and has demonstrated reliable diagnostic value in previous studies focusing on acid reflux. However, over the past decade, the significance of non-acid reflux in gastroesophageal reflux has gradually gained attention. Particularly, among patients receiving acid-suppressive therapy, the occurrence rate of non-acid reflux can reach up to 80% [33, 34], leading to persistent cough symptoms that hinder recovery [35]. This highlights the limitations of AET. SI and SAP serve as supplementary parameters for establishing the relationship between reflux and cough, aiding in the diagnosis of GERC. However, their acquisition relies on patients documenting the timing of cough events in a diary card, which greatly influences their accuracy. Research conducted by Kavitt et al. [36] indicated that within a 2-minute time window, 74–91% of cough events were missed. Slaughter [11] suggested that SI and SAP are unreliable when the reflux rate is low, as their positive results are susceptible to chance and exhibit high daily variability. Consequently, their actual clinical significance is often overinterpreted. Therefore, the existing parameters such as AET, SI, and SAP are insufficient to meet the clinical diagnostic needs of GERC, necessitating further optimization through the incorporation of new reliable parameters.

PSPWI and MNBI are novel parameters derived from the MII-pH technology. Their application is based on the pathophysiological mechanisms of GERD and have been increasingly recognized in the diagnosis and treatment of GERD in recent years. GERD is a complex disease that is typically believed to be caused by multiple factors [29, 37]. Currently, the pathogenesis of GERD is generally considered to be associated with LES hypotension, transient LES relaxation, hiatal hernia, impaired esophageal clearance, acid pocket formation, esophageal hypersensitivity, and mucosal injury [37–39]. Esophageal clearance function serves as a protective mechanism for the esophagus. During reflux episodes, volume clearance is facilitated by secondary peristaltic waves, which clear approximately 90% of the refluxate [40]. Chemical clearance is induced by swallowing, facilitating the transport of bicarbonate and epidermal growth factor from saliva, restoring the pH of the distal esophagus, and repairing damaged mucosa [41]. A low PSPWI indicates impaired esophageal clearance, leading to prolonged exposure of the distal esophagus to refluxate and resulting in mucosal integrity damage [12], cell-to-cell space dilation [42], and increased paracellular permeability of epithelial cells, manifested as a decrease in baseline impedance [13]. Previous studies have shown that PSPWI and MNBI improve the accuracy of GERD diagnosis using MII-pH monitoring [15], help us to distinguish non-erosive reflux disease (NERD) from functional heartburn patients [21, 43], and may serve as reliable parameters for differentiating GERD phenotypes. Kessing et al. [44] demonstrated a correlation between baseline impedance values and esophageal acid exposure, and observed lower distal MNBI than proximal MNBI in patients with gastroesophageal reflux, which may be associated with more severe reflux of gastric contents into the distal esophagus. This is consistent with our data results.

In our study, we have observed an interesting finding which suggests that the combined use of AET and PSPWI significantly improves the diagnosis of non-acid GERC, as it has been able to identify over 80% of non-acid GERC patients, thereby aiding in the diagnosis of GERC. Non-acid reflux accounts for up to 40% of all gastroesophageal reflux cases [45], and it is often difficult to diagnose non-acid GERC using AET and SAP. In a systematic review, it was found that among patients with refractory GERD to PPIs, non-acid reflux accounted for up to 63% of all cases, and over 80% of reflux-related symptoms were associated with weakly acid or alkaline reflux [33]. While PPIs can reduce the acidity of refluxate, they are not effective in reducing the volume and frequency of refluxate, making them unsuitable for the treatment of non-acid GERC. Patients with this type of GERC require further management, including the use of drugs such as baclofen [46, 47] to alleviate reflux and cough symptoms. However, these patients are often misdiagnosed or undiagnosed, leading to delays in receiving appropriate treatment and prolonged suffering. Hence, the use of PSPWI appears to be promising in addressing this issue and can potentially aid in the diagnosis and treatment of GERC. We intend to further explore this in the future studies.

In our study, we observed that PSPWI demonstrated lower levels in patients with GERC as compared to non-GERC patients. This observation indicates that PSPWI holds diagnostic value for GERC and helps compensate for the limitations of AET in identifying GERC patients with predominant non-acid reflux. Furthermore, the combination of PSPWI with AET further enhances the diagnostic value for GERC. On the other hand, the application of MNBI in diagnosing GERD appears to be less effective compared to PSPWI. However, MNBI does offer advantages in terms of a simpler data collection process and provides some degree of reference value for the diagnosis of GERC. Of particular importance, both PSPWI and MNBI exhibit advantages in predicting the efficacy of anti-reflux treatment. The main treatment approach for GERC typically involves the use of acid-suppressing medications alone or in combination with prokinetic agents, with PPIs being the preferred choice [37, 48]. However, approximately one-third of patients show poor response to PPI therapy, indicating refractory GERC [34]. Existing evidence suggests that MNBI values are significantly lower in non-responders to PPI or anti-reflux treatment compared to responders [23, 49], and MNBI can predict the response of patients with typical reflux symptoms to PPI or anti-reflux treatment [50, 51]. Frazzoni et al. [52] found that MNBI and PSPWI provide better predictions of symptom response to PPI therapy compared to AET. Studies have also shown that MNBI has good predictive value in the standardized management of non-acid GERC [53]. Therefore, we believe that the practical significance of PSPWI and MNBI extends beyond these findings and calls for further exploration, as they may become essential components in the diagnostic and therapeutic processes of GERC in the future.

We acknowledge that our study has certain limitations. We conducted a retrospective study involving a strictly selected group of clinical cases and utilized corresponding MII-pH data for statistical analysis. However, we did not provide a systematic evaluation of macroscopic or microscopic changes in the esophageal mucosa, nor did we assess esophageal motility changes. In the future, we intend to conduct comprehensive research with a prospective study design to further investigate these aspects in-depth.

## Conclusion

In general, PSPWI is a parameter with significant diagnostic value for GERC. We recommend the combined use of PSPWI < 39.4% or AET > 4.4% for diagnosing GERC to reduce the rate of missed non-acid GERC diagnoses by approximately 44%. MNBI and distal MNBI, while less sensitive, can still serve as useful reference indicators in diagnosing GERD, particularly when PSPWI data is lacking.

## Data Availability

No datasets were generated or analysed during the current study.
